# Systematic review of externally validated machine learning models for predicting acute kidney injury in general hospital patients

**DOI:** 10.3389/fneph.2023.1220214

**Published:** 2023-08-03

**Authors:** Marina Wainstein, Emily Flanagan, David W. Johnson, Sally Shrapnel

**Affiliations:** ^1^ Faculty of Medicine, University of Queensland, Brisbane, QLD, Australia; ^2^ Department of Medicine, West Moreton Kidney Health Service, Ipswich Hospital, Brisbane, QLD, Australia; ^3^ Faculty of Science, University of Queensland, Brisbane, QLD, Australia; ^4^ Metro South Kidney and Transplant Services (MSKATS), Princess Alexandra Hospital, Brisbane, QLD, Australia; ^5^ Centre for Kidney Disease Research, University of Queensland at Princess Alexandra Hospital, Brisbane, QLD, Australia; ^6^ Centre for Kidney Disease Research, Translational Research Institute, Brisbane, QLD, Australia; ^7^ Centre for Health Services Research, University of Queensland, Brisbane, QLD, Australia; ^8^ Australian Research Council (ARC) Centre of Excellence for Engineered Quantum Systems, School of Mathematics and Physics, University of Queensland, Brisbane, QLD, Australia

**Keywords:** systematic review, acute kidney injury, AKI, machine learning, prediction, risk score, models

## Abstract

Acute kidney injury (AKI) is one of the most common and consequential complications among hospitalized patients. Timely AKI risk prediction may allow simple interventions that can minimize or avoid the harm associated with its development. Given the multifactorial and complex etiology of AKI, machine learning (ML) models may be best placed to process the available health data to generate accurate and timely predictions. Accordingly, we searched the literature for externally validated ML models developed from general hospital populations using the current definition of AKI. Of 889 studies screened, only three were retrieved that fit these criteria. While most models performed well and had a sound methodological approach, the main concerns relate to their development and validation in populations with limited diversity, comparable digital ecosystems, use of a vast number of predictor variables and over-reliance on an easily accessible biomarker of kidney injury. These are potentially critical limitations to their applicability in diverse socioeconomic and cultural settings, prompting a need for simpler, more transportable prediction models which can offer a competitive advantage over the current tools used to predict and diagnose AKI.

## Introduction

An estimated one in five adults admitted to hospital develop acute kidney injury (AKI) (1). Acute kidney injury is associated with an increased risk of in-hospital death and long-term development of chronic kidney disease (CDK) and other non-kidney complications, such as HTN, cardiovascular disease and stroke (2).

Multiple prediction models and risk scores have been developed over the years to predict the occurrence of hospital-acquired AKI (HA-AKI) in order to improve short- and long-term management (3). In patients presenting to the emergency department (ED) these interventions may be as simple as performing a fluid assessment to optimize hydration status and avoiding nephrotoxic medications. AKI prediction models or risk scores have been developed for various populations, from undifferentiated general hospital patients to critically ill or post-operative patients. Accordingly, predictors used have ranged from general admission observations to laboratory data and specific procedural interventions. Acute kidney injury is most commonly defined using the Kidney Disease Improving Global Outcomes (KDIGO) criteria which uses the rise in serum creatinine or decrease in urine output (4), however, other definitions - such RIFLE, AKIN - have been used in AKI models.

Over the past decade as larger and more complex patient databases have become available, development of machine learning (ML) models for risk prediction has become increasingly popular. Compared to traditional regression models, ML models have the advantage of not being constrained by assumptions between variables and outcomes allowing more subtle relationships in the data to be explored. Additionally, their computational power means that a greater number of input variables and data points can be processed to make more accurate and individualized forecasts.

Past systematic reviews of prediction models in AKI have identified a high risk of bias in most studies resulting from variability in the use of performance measures (in particular calibration reporting), sparse interpretability considerations, possibility of overfitting, narrow scope for reproducibility and a lack of external validation limiting the generalizability and applicability of the models (3, 5). Moreover, while AKI risk prediction is useful in sub-specialized patient populations, the majority of AKI occurs in the general wards, placing a premium on flexible risk prediction models that can perform well in these settings (6).

Hence, the research question of this systematic review is: what are the available machine learning models which can assist a health professional to quantify the risk of KDIGO-defined AKI during a hospital stay in patients admitted to a general hospital or clinic? Accordingly, the aim of this systematic review was to evaluate and critically appraise studies of models developed from general hospital populations, using the current KDIGO definition of AKI, and which have been externally validated.

## Methods

This review was designed and conducted according to Preferred Reporting Items and Systematic Review and Meta-Analyses (PRISMA) Protocol guideline (7) and the Checklist for critical Appraisal and data extraction for systematic Reviews of prediction Modelling Studies (CHARMS) (8).

### Study identification

We used PubMed (pubmed.ncbi.nlm.nih.gov) and the Institute of Electrical and Electronic Engineers (IEEE) *Xplore* Digital library (https://ieeexplore.ieee.org/Xplore/home.jsp) for our search. In PubMed we searched titles or abstract with a combination of string and Mesh terms (**Table S1**). The search was conducted on July 13^th^, 2022 and was limited to studies published in the last five years (up to July 13^th^ 2017).

### Study inclusion and selection

We included studies that validated machine learning models, with or without initially developing those models, for the prediction of AKI (stage 1, 2, 3 or dialysis) in a general hospital population. We excluded studies using the following criteria:

- studies in patients under 18 years of age;
- studies which used an outcome definition of AKI other than KDIGO;
- studies presenting models based on regression, such as linear, logistic or penalized regression, or those which are well understood theoretically and by clinicians, such as Naïve Bayes;
- studies presenting models with no external validation;
- any comments or editorials or meta-analysis or systematic reviews.

We considered external validation to exist if the developed model was applied to new individuals, either from the same institution but a different time period (temporal validation), from a different institution or country (geographic validation) or on very different individuals (domain validation) (9).

Titles and abstracts were screened by two reviewers (MW and EE), and full articles were reviewed if they were eligible or if reviewers disagreed in the screening process. Disagreements were resolved by a third reviewer (SS).

### Data extraction and critical appraisal

A data extraction form was created based on the Checklist for Critical Appraisal and Data Extraction for Systematic Reviews of Prediction Modelling Studies (CHARMS) checklist (8) and the Transparent Reporting of a multivariable prediction model for Individual Prognosis Or Diagnosis (TRIPOD) (10). Items extracted included study type, number and location of centers recruited from, patient characteristics (AKI incidence, staging and mortality), model characteristics including prediction windows, predictors used, types of models evaluated, type of external validation performed, method used to calculate baseline serum creatinine and metrics used to evaluate the models among others. Risk of bias was assessed using the Prediction Model Risk of Bias Assessment Tool (PROBAST), which includes four domains and 20 signaling questions (11). Domains were rated as high risk, low risk or unclear risk. Models would need be low risk on all domains to be evaluated overall as low risk. It should be noted that this tool was created for traditional regression models and is currently being updated to include newer model methodology. Data extraction and appraisal were done by EE and MW. Disagreements were resolved through discussion with SS.

## Results

**Figure 1:**
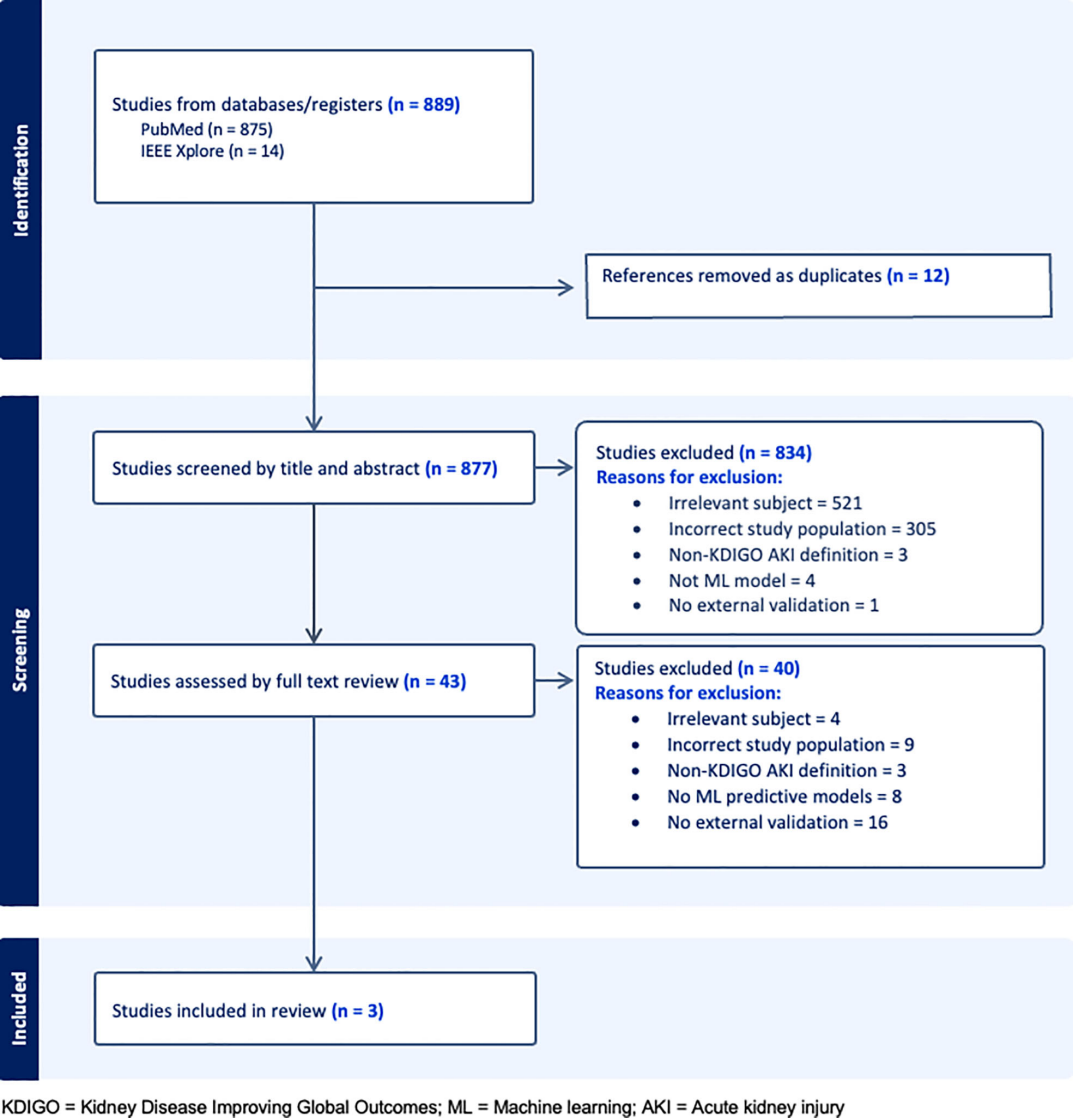
PRISMA flow diagram.

Our search returned 889 studies. A majority of studies were excluded initially either because they did not present or validate a prediction model (this included commentaries, editorial letters, narrative reviews and systematic reviews), the subject was irrelevant and/or the population studied was not comprised of general hospital patients (**Figure 1**). Forty-three were selected for full text review either because they fit the inclusion criteria or because insufficient information was given in the title or abstract to decide on eligibility. A further 40 studies were excluded following full text review of which 16 were excluded for not having external validation. Of the remaining three studies, two developed and validated a model (1 and 3) and one validated a previously developed model (12) (study 2). A list of the types of models excluded based on criteria (iii) can be seen in **Table S2**.

### General study characteristics

#### Data source and population characteristics

All studies were from urban or suburban tertiary, referral centers in high income countries, two from the United States and one from South Korea (**Table 1**). Data was sourced either from the individual hospital electronic health records (EHR) (studies 1 and 2) or from clinical research networks, such as the Greater Plains Collaborative (GPC) (study 3). Studies included hospitalized patients with at least two documented serum creatinine measures and/or a minimum of 48 hours of admission (studies 1 and 3) Patients with severe forms of AKI or on kidney replacement therapy (KRT) were uniformly excluded (**Data Sheet 1**). Patient data was variably collected between 2006 and 2018.

**Table 1:** Study population characteristics.

	Study 1 (13)	Study 2 (14)	Study 3 (15)
**Number & type of sites**	SNUBH: tertiary hospital SNUH: tertiary hospital	UC: urban, tertiary hospital LUMC: suburban tertiary hospital NUS: suburban, 4-hospital health care network	6 GPC sites from 5 US states (all tertiary, referral centers)
**Country of sites**	Korea	USA	USA
**Type of data & collection**	EHR/Retrospective	EHR/Retrospective	EHR/Retrospective
**Population setting**	General inpatients	ED, wards and ICU	Inpatients
**Participant eligibility**	Age > 18 yo Hospitalized for more than 48 hrs	Age > 18 yo	Patients aged 18 - 90 years old Hospitalized for more than 48 hrs At least 2 sCr
**Population size**	SNUBH = 76,756 SNUH = 72,352	UC = 48,463 LUMC = 200,613 NUS = 246,895	Site 1: 153,821 encounters Site 2: 100,819 encounters Site 3: 86,264 encounters Site 4: 57,286 encounters Site 5: 19,542 encounters Site 6: 88,865 encounters
**Time period data collection**	2013-2017	UC = 2008 – 2016 LUMC = 2007 – 2017 NUS = 2006 - 2015	Jan 1^st^ 2010 to 31^st^ December 2018
**Experimental design**	SNUBH = 90% T, 10% IV SNUH = EV	UC = IV LUMC & NUS = EV	Site 1: Encounters from 2010 to 31^st^ Dec 2016 = 70% T, 15% C, 15% IV. Encounters Jan 2017 onwards: EV Sites 2 – 6: EV
**Female (%)**	SNUBH = 46.5% SNUH = 48.3%	UC = 53.5% NUS = 56.7% LUMC = 50.3%	All sites: 44 – 52.5%
**Age (median/mean)**	SNUBH = 59.6 SNUH = 57.1	UC = 56.6 LUMC = 58.6 NUS = 67.4	% of patients ≥ 66 yo: 21 – 60.2%
**Minority representation**	Not reported	African American UC = 50% LUMC 22.7% NUS = 7.3%	All sites Black = 0.4 – 21% Asian = 0.5 – 1.9% Native American = 0 – 1% Hispanic = 0.4 – 11.5%
**AKI incidence**	SNUBH = 4536 (5.91%) SNUH = 2626 (3.63%)	Any AKI = 8.3 – 14.3% AKI ≥ Stage 2 UC = 1664 (3.4%) LUMC = 5722 (2.8%) NUS = 3499 (1.4%)	All sites: Any AKI: 10.1 – 16% Stage ≥ 2 AKI: 1.6 – 3.2% Stage 3 AKI: 0.6 – 1.9%

AKI, Acute kidney injury; EHR, Electronic health records; ED, Emergency department; GPC, Greater Plains Collaborative; ICU, Intensive Care Unit; sCr, serum creatinine; eGFR, estimate glomerular filtration rate; RRT, renal replacement therapy; SNUBH, Seoul National University Bundang Hospital; SNUH, Seoul National University Hospital; UC, University of Chicago; LUMC, Loyola University Medical Center; NUS, Northshore University Health System; T, training; C, calibration; IV, internal validation; EV, external validation.

The experimental design used to develop and/or validate model predictions varied between studies. Study 1 split the population of one hospital into training and internal validation cohorts, and the population of the second hospital, from the same city, was used for external validation. Study 2 was based on a previously developed model which was internally validated in the original hospital population and externally validated in a four-hospital health care network and a fifth hospital from the same state. Study 3 used the encounters from site 1 from 2010 to 2016 for training, calibration and internal validation. It then used the encounters from 2017 to 2018 and the population of five other GPC sites for external validation.

All validation cohorts had close or over 50,000 patients, except for site 5 in study 3 which had 19,542. Gender distribution was even in all studies. The median or average age was close to 60 in most cohorts with study 3 exhibiting wider variability between the six sites. Black or African American patients comprised 50% of the internal validation cohort of study 2 and up to 20% in the remaining US study. Only study 3 reported the prevalence of Hispanic, Native American and Asian patients, who accounted for no more than 10% of all patients. Incidence of AKI was lowest in study 1 (3-6%), followed by study 2 (8.3 – 14.3%), and study 3 (15.1%).

#### Outcome

Acute kidney injury was defined by the serum creatinine criteria of the KDIGO definition in all studies. Studies 1 and 3 used AKI of any grade as the primary outcome while study 2 predicted AKI stage 2 or higher (**Table 2**). All studies used a 48-hr prediction window except for study 1 that predicted AKI within seven days of the present. Prediction was continuous throughout a patient’s admission in all studies with data binned into time windows between 6 to 24 hrs. Two of three studies (1 and 3) prioritized a pre-admission serum creatinine to represent the baseline value, followed by admission measurements (study 2) or imputation using the Modification of Diet in Renal Disease (MDRD).

**Table 2:** Prediction model characteristics and results.

	Study 1 (13)	Study 2 (14)	Study 3 (15)
**Primary prediction outcome**	AKI within 7 days	AKI Stage ≥ 2 within 48 hrs	AKI within 48 hrs
**Method used to calculate baseline sCr**	1) Min sCr within 2 weeks pre-admission, or 2) Min sCr within 90-180 days pre-admission, or 3) Admission sCr	Admission sCr	1) Most recent sCr pre-admission, or 2) First sCr on admission
**Static vs Continuous prediction**	Continuous	Continuous	Continuous
**Number of predictors in final model**	107	59	1933
**Predictor categories in final model**	Demographic, vital signs, comorbidities, medications, laboratory values and clinical conditions (e.g., ICU admission)	Demographic, absolute values and trend information for vital signs and laboratory values, interventions, medications, procedures, LOS, nursing documentation (e.g., Braden score).	Demographic, diagnoses, procedures, lab tests, medications, vital signs
**Method for predictor variable selection**	Features considered as risk factors of AKI from the literature or correlated with AKI development.	Not mentioned	All variables in the PCORnet CDM schema shared across the 6 GPC sites
**Best performing model type**	RNN	DS-GBM	DS-GBM
**Comparison model**	GBM	–	1) LASSO model 2) Model (GBM & LASSO) without sCr and BUN data
**External validation** **performance AUROC** 1 = Any AKI 2 = Stage ≥ 2 AKI	SNUH = 1) 0.84, 2) 0.90	LUMC = 2) 0.85 NUS = 2) 0.86	Site 1: 1) 0.76, 2) 0.81 Site 2: 1) 0.75, 2) 0.79 Site 3: 1) 0.63, 2) 0.73 Site 4: 1) 0.6, 2) 0.68 Site 5: 1) 0.71, 2) 0.8 Site 6: 1) 0.62, 2) 0.71
**Other performance measures**	Sensitivity, specificity, PPV, NPV, F1 score, MSE	Calibration using visual plots Sensitivity, specificity, PPV, NPV	AUPRC Calibration using Hosmer- Lemeshow score
**Type of external validation**	Geographic: SNUH	Geographic: LUMC & NUS	Temporal: site 1 Geographic: sites 2-6
**Interpretability**	1) Shapley Additive Explanations, partial Dependence Plots (PDP) and accumulated local effects plots 2) Instance-wise interpretation with individual conditional expectation (ICE) plots	Variable importance plot	Shapley Additive Explanations
**Top predictive variables**	1) Baseline eGFR 2) Baseline sCr	1) Change in sCr 2) Length of stay 3) SF ratio	1) sCr and its change 2) Vancomycin exposure 3) Minimal value & hourly change in BP value
**Handling of missing information**	Carry-forward imputation	Median (for continuous data) or mode (for categorical data) by location being imputed for missing predictor values that remained after carry-forward imputation	Carry-forward imputation
**Proportion of missing data**	Not reported	Not reported	Not reported
**Code availability**	Not provided	Not provided	Available on GitHub

sCr, serum creatinine; MDRD, Modification of Diet in Renal Disease; RNN, Recurrent neural network; DS-GBM, Gradient Boosting Model with discrete time survival framework; ML, machine learning; AUROC, area under the receiver operating curve; AUPRC, area under the precision receiver curve; PPV, positive predictive value, NPV, negative predictive value, MSW, mean squared error; SNUH, Seoul National University Hospital; SNUH, Seoul National University Hospital; UC, University of Chicago, LUMC, Loyola University Medical Center; NUS, Northshore University Health System; LASSO, Least absolute shrinkage and Selection Operator; CDM, common data model; SF ratio, ratio of oxygen saturation in arterial blood to the % of oxygen in inspired air.

### General model characteristics

#### Predictor data

The lowest number of predictor variables was 59 (study 2) and the highest was 1933 (study 3) (**Table 2**). Study 3 used all available variables in their health system or network EHR, while study 1 selected those known to be associated with AKI risk and study 2 did not specify the variable selection process. Most variable categories included demographic information, vital signs, laboratory and diagnostic data, comorbidities, medications, clinical conditions (usually labelled by ICD 9 or 10 codes) procedures or interventions as well as nursing documentation (study 2) and length of stay. All studies included sCr and blood urea nitrogen (BUN) as predictors but only study 3 evaluated model performance with and without the inclusion of these variables. All studies used carry-forward imputation for missing data but none of them reported the actual proportion of missing data.

#### Type of prediction models and performance

In the external validation cohorts and for the prediction of stage 2 or higher AKI within 48 hrs, Study 2, which used a Gradient Boosting Model with discrete time survival framework (DS-GBM) had the best performing model (AUROC 0.85-0.86) followed by study 3 (AUROC 0.68 – 0.81), which also used DS-GBM. Study 1, which used a Recurrent Neural Network (RNN) to predict AKI within 7 days had excellent performance (AUROC all AKI 0.84 and stage ≥ 2 AKI 0.90). Performance was compared to a GBM in study 1 and to a Least Absolute Shrinkage and Selection Operator (LASSO) model in study 3.

Beyond discrimination, studies evaluated models using calibration (1 and 2), sensitivity, specificity, positive and negative predictive value (1-3), area under the precision receiver curve (3) and F1 score (1). All studies performed some form of interpretability analysis to contextualize predictions which showed that serum creatinine – either as a baseline absolute value, in the form of change over time or integrated within an eGFR equation - was consistently the strongest predictor of future AKI risk. Further study and model information can be found in **Data Sheet 1** of the **Supplemental Material**.

#### Type of external model validation

All three studies performed geographic external validation and study 3 also performed temporal validation using the later encounters from the source population.

### Critical appraisal

#### Assessment of bias, applicability and reproducibility

All except for study 3 were found to have a high risk of bias in the participant and outcome domains. The former resulting from the use of retrospective EHRs as primary data source and the latter to the incorporation bias inherent in the inclusion of serum creatinine in the outcome definition. We considered the development of a second model without serum creatinine and BUN information in Study 3, as an attempt to mitigate this bias. The predictors and analysis domains were uniformly deemed as having low risk of bias (**Table 3**). Applicability was a concern for studies 1 and 3 that used more than 100 predictor variables. With regards to reproducibility, only study 3 shared code in a publicly accessible domain. Individual study PROBAST assessment forms can be found in the **Supplemental Material**.

**Table 3:** Traffic light plot of risk of bias assessment.

		Study 1	Study 2	Study 3
**ROB**	Participants	high	high	?
	Predictors	low	low	low
	Outcome	high	high	low
	Analysis	low	low	low
**Applicability**	Participants	low	low	low
	Predictors	high	low	high
	Outcome	low	low	low
**Overall**	ROB	high	high	low
	Applicability	high	low	low

ROB, risk of bias; ?, unclear risk.Red = high risk, Green = low risk, Yellow = Unclear risk.

## Discussion

We screened 889 studies from the last five years in search of externally validated ML prediction models of AKI based on general hospital patients. Only three studies were retrieved that fit these criteria. While most performed well and had a sound methodological approach, the main concerns relate to their development and validation in populations with limited diversity, comparable digital ecosystems and use of large number of predictor variables. These are potentially critical limitations to their applicability in diverse socioeconomic and cultural settings, prompting a need for simpler and more transportable prediction models. In addition, all models rely heavily on serum creatinine, which questions their added utility and advantage over the current tools used to predict and diagnose AKI.

Of the 886 studies not included, a majority were excluded due to lack of external validation, use of sub-populations (mainly ICU and surgical cohorts) or predictions based on standard regression, parametric models. The finding of a growing number of prediction models generated on clinical sub-populations, often from publicly available datasets, without any external validation has been noted by previous systematic reviews (3, 5).

Most models performed well, especially when predicting stage 2 or higher AKI, which is often regarded as the more meaningful outcome in the acute setting (16). Discrimination remained high between internal and external validation sites in studies 1 and 2 but varied widely across the 6 sites of study 3 which had the highest diversity in demographic profiles and EHR structures. The main barriers to independent validation of a prediction model include patient heterogeneity, clinical process variability, EHR configuration and data warehouse heterogeneity leading to non-interoperable databases across hospitals (15). Models should be ideally trained and validated on diverse populations from healthcare sites with a spectrum of maturity and sophistication in their digital ecosystems. There is little point in training a model with thousands of variables that it is unlikely to ever encounter when applied to a different healthcare system. Hence, the concerns of applicability in most of the presented studies. To address this, Song et al. (study 3) explored the source of performance variability between the original and target sites and proposed a metric for model transportability. Interestingly, the study brings to light how differences in data representation and format between individual EHRs can have a significant impact on what a model learns and how it performs (15). Overall, this would suggest that a model which uses fewer and more commonly available clinical variables, like the one from study 2, is likely to have better uptake and be more easily validated.

From applicability to implementation, there is a growing concern that much of the attention in the field of machine learning risk prediction has centered around model development and not on the transition to implementation and deployment (17). Providing a framework on the lifecycle management of a model is critical to its success as a clinical decision support tool. This includes information on how often a model should be re-validated, potentially re-trained and fine-tuned as well as how it should be integrated into clinical workflows (18, 19). While this information may be available in the coming years, at present none of the studies specifically addressed this. It should also be said that the reluctance of most groups to share their development code, hinders efforts to make the models more reproducible and transportable.

The use of serum creatinine as a predictor and as part of the outcome definition is problematic. The latter results in incorporation bias whereby the association between the predictor and the outcome is overestimated rendering model performance evaluations optimistic (11). In reality, however, any model that uses the current serum creatinine based KDIGO definition of AKI and has access to repeated measures of serum creatinine, will suffer from this bias. With regards to the use of creatinine as a predictor, the interpretability analyses of all studies revealed that either the baseline serum creatinine and eGFR (which includes serum creatinine in its calculation) or the change in serum creatinine over time were among the top three heaviest contributors to the prediction. The question then becomes: *what can these models offer that a single or trend in serum creatinine readings cannot?* Study 3 trained a model without any information on serum creatinine or BUN which performed competitively with an AUROC of 0.75 for any AKI and 0.82 for stage 2 or higher AKI. The main predictors of AKI in this model were vancomycin exposure, blood pressure change, age, BMI, height, having a chest x-ray and receiving Tazosin antibiotic. It should be noted, however, that this model used almost 2000 predictor variables to arrive at this prediction.

Perhaps the real advantage of these models lies not in their capacity to process large and complex health data in order make timely predictions but in their potential for integration within clinical workflows. A health professional must know to order a serum creatinine test on a particular at-risk patient, they must then remember to check it once it has been reported and to act on it using evidence-based guidelines. A disruption to this process, which is time-consuming and often falls to the most junior medical staff in the hospital, may result in a missed opportunity to avoid or minimize the severity and consequences of AKI. Automating this process with a prediction algorithm that can seamlessly aggregate the relevant historical and admission information and present the clinician with a real-time risk profile for each patient on any given day, may go a long way towards decreasing the incidence of hospital-acquired AKI. It remains to be seen, however, whether the same can be achieved with more comprehensive education of junior doctors and clearer accountability structures within healthcare teams.

The main limitation of our study is the narrow search and selection criteria. In wanting to explore only machine learning as a novel technology in AKI risk prediction we have not compared or evaluated the more traditional regression models and risk scores, such as linear and logistic regression or Naïve Bayes classifier. It would be prudent to say that, at this stage, the benefits of ML in AKI are not clear. Relying on more explainable, less complex statistical models while this technology matures, may be the wiser approach. In addition, by selecting only models that had been externally validated we may have missed studies with sound methodology that were in the early stages of validation.

The use of ML models for AKI prediction requires large and complex data structures to support them, have potentially limited applicability beyond the health systems they were trained on and, at present, rely heavily on a cheap and readily accessible biomarker of kidney injury with little apparent benefit from the other predictors. In the future, simpler and more transportable models that offer a significant predictive advantage to using serum creatinine measurements may find greater clinical utility and acceptance within the healthcare community.

## Data availability statement

The original contributions presented in the study are included in the article/**Supplementary Material**. Further inquiries can be directed to the corresponding authors.

## Author contributions

MW and SS were responsible for the conception and design of this systematic review. EF and MW performed in equal parts the acquisition, analysis, interpretation and presentation of the data. MW drafted the manuscript. DJ and SS revised each draft critically for important intellectual content. All authors agree to be accountable for all aspects of the work in ensuring that questions related to the accuracy or integrity of any part of the work are appropriately investigated and resolved. All authors contributed to the article and approved the submitted version.

## References

[1] Susantitaphong P Cruz DN Cerda J Abulfaraj M Alqahtani F Koulouridis I . World incidence of AKI: A meta-analysis. Clin J Am Soc Nephrol (2013) 8(9):1482–93. doi: 10.2215/cjn.00710113 PMC3805065 23744003

[2] Kellum JA Romagnani P Ashuntantang G Ronco C Zarbock A Anders H-J . Acute kidney injury. Nat Rev Dis Primers (2021) 7(1):52. doi: 10.1038/s41572-021-00284-z 34267223

[3] Hodgson LE Sarnowski A Roderick PJ Dimitrov BD Venn RM Forni LG . Systematic review of prognostic prediction models for acute kidney injury (AKI) in general hospital populations. BMJ Open (2017) 7(9):e016591. doi: 10.1136/bmjopen-2017-016591 PMC5623486 28963291

[4] Kidney Disease: Improving Global Outcomes (KDIGO) Acute Kidney Injury Workgroup . KDIGO Clinical practice guideline for acute kidney injury. Kidney Int Suppl (2011) (2012) 2(1):1–138. doi: 10.1038/kisup.2012.1

[5] Vagliano I Chesnaye NC Leopold JH Jager KJ Abu-Hanna A Schut MC . Machine learning models for predicting acute kidney injury: a systematic review and critical appraisal. Clin Kidney J (2022) 15(12):2266–80. doi: 10.1093/ckj/sfac181 PMC9664575 36381375

[6] Koyner JL Cerdá J Goldstein SL Jaber BL Liu KD Shea JA . The daily burden of acute kidney injury: a survey of US nephrologists on World Kidney Day. Am J Kidney Dis (2014) 64(3):394–401. doi: 10.1053/j.ajkd.2014.03.018 24815216

[7] Moher D Liberati A Tetzlaff J Altman DG . Preferred reporting items for systematic reviews and meta-analyses: the PRISMA statement. J Clin Epidemiol (2009) 62(10):1006–12. doi: 10.1016/j.jclinepi.2009.06.005 19631508

[8] Moons KG de Groot JA Bouwmeester W Vergouwe Y Mallett S Altman DG . Critical appraisal and data extraction for systematic reviews of prediction modelling studies: the CHARMS checklist. PloS Med Oct (2014) 11(10):e1001744. doi: 10.1371/journal.pmed.1001744 PMC4196729 25314315

[9] Moons KG Kengne AP Grobbee DE Royston P Vergouwe Y Altman DG . Risk prediction models: II. External validation, model updating, and impact assessment. Heart (2012) 98(9):691–8. doi: 10.1136/heartjnl-2011-301247 22397946

[10] Collins GS Reitsma JB Altman DG Moons KGM . Transparent reporting of a multivariable prediction model for individual prognosis or diagnosis (TRIPOD): the TRIPOD statement. BMJ: Br Med J (2015) 350:g7594. doi: 10.1136/bmj.g7594 25569120

[11] Moons KGM Wolff RF Riley RD Whiting PF Westwood M Collins GS . PROBAST: A tool to assess risk of bias and applicability of prediction model studies: explanation and elaboration. Ann Intern Med (2019) 170(1):W1–W33. doi: 10.7326/M18-1377 30596876

[12] Koyner JL Carey KA Edelson DP Churpek MM . The development of a machine learning inpatient acute kidney injury prediction model*. Crit Care Med (2018) 46(7):1070–7. doi: 10.1097/ccm.0000000000003123 29596073

[13] Kim K Yang H Yi J Son H-E Ryu J-Y Kim YC . Real-time clinical decision support based on recurrent neural networks for in-hospital acute kidney injury: External validation and model interpretation. J Med Internet Res (2021) 23(4):e24120. doi: 10.2196/24120 33861200 PMC8087972

[14] Churpek MM Carey KA Edelson DP Singh T Astor BC Gilbert ER . Internal and external validation of a machine learning risk score for acute kidney injury. JAMA Netw Open (2020) 3(8):e2012892. doi: 10.1001/jamanetworkopen.2020.12892 32780123 PMC7420241

[15] Song X Yu AS Kellum JA Waitman LR Matheny ME Simpson SQ . Cross-site transportability of an explainable artificial intelligence model for acute kidney injury prediction. Nat Commun (2020) 11(1):5668. doi: 10.1038/s41467-020-19551-w 33168827 PMC7653032

[16] Murray PT Mehta RL Shaw A Ronco C Endre Z Kellum JA . Potential use of biomarkers in acute kidney injury: report and summary of recommendations from the 10th Acute Dialysis Quality Initiative consensus conference. Kidney Int (2014) 85(3):513–21. doi: 10.1038/ki.2013.374 PMC4198530 24107851

[17] Hofer IS Burns M Kendale S Wanderer JP . Realistically integrating machine learning into clinical practice: a road map of opportunities, challenges, and a potential future. Anesth Analg (2020) 130(5):1115. doi: 10.1213/ANE.0000000000004575 32287118 PMC7584400

[18] Joshi M Mecklai K Rozenblum R Samal L . Implementation approaches and barriers for rule-based and machine learning-based sepsis risk prediction tools: a qualitative study. JAMIA Open (2022) 5(2):ooac022. doi: 10.1093/jamiaopen/ooac022 35474719 PMC9030109

[19] Marwaha JS Landman AB Brat GA Dunn T Gordon WJ . Deploying digital health tools within large, complex health systems: key considerations for adoption and implementation. NPJ Digital Med (2022) 5(1):13. doi: 10.1038/s41746-022-00557-1 PMC8795422 35087160

