# Supplementary material for: Systematic review of externally validated machine learning models for predicting acute kidney injury in general hospital patients
Source: Front Nephrol. 2023 Aug 3;3:1220214. doi: 10.3389/fneph.2023.1220214 (PMC10479567; doi:10.3389/fneph.2023.1220214)
Supplement: Supplementary file 2 [file Table_1.docx]

Supplementary material

**Table S1** Search criteria

| **PubMed** | "Acute Kidney Injury"[Mesh] OR “acute kidney injury”[tiab] OR AKI[tiab] OR “acute renal injury”[tiab] OR “acute renal failure”[tiab] OR ARF[tiab] OR “Contrast induced nephropathy”[tiab] OR (contrast[tiab] AND nephropathy[tiab]) OR (( Acute[tiab] OR injury[tiab]) AND ("Renal Dialysis"[Mesh] OR renal dialysis[tiab] OR hemodialysis[tiab] OR haemodialysis[tiab])) OR “renal replacement therap*”[ti]  AND  "neural networks, computer"[MeSH Terms] OR "machine learning"[mesh] OR "machine learning"[tiab] OR "neural network*"[tiab] OR "artificial intelligence"[tiab] OR "Artificial Intelligence"[Mesh] OR computer simulation[tiab] OR "Computer Simulation"[mesh] OR statistical learning[tiab] OR "Linear Models"[Majr] OR "Linear regression"[ti] OR "Linear model"[ti] OR "K-nearest neighbo*" OR "Support vector machine" OR "Radial basis function" OR "Naïve bayes" OR "Decision tree classification" OR "Random forest*" OR "Clinical decision support" OR “Supervised learning” OR “Unsupervised learning” OR “Deep learning algorithms” OR “Neural networks” OR “data mining” OR “Integrated probabilistic model*” OR “Random forest model” OR “classification tree analysis” OR "predictive analytic*" OR "predictive algorithm*" OR "risk score"[tiab] OR "prediction model*" OR “predictive model*” OR "risk prediction" OR "deep learning" OR "logistic regression"[ti] OR "Natural Language Processing"[Mesh] OR "Natural Language Processing" OR "NLP" OR "Deep Learning"[Mesh] OR "XGBoost" OR "AdaBoost" OR "boosted tree*" OR "support vector machine*" OR "gradient boosting" OR "decision-tree*" OR "decision tree*" OR "Quadratic Discriminant Analysis" OR "Linear Discriminant Analysis" OR "principal components analysis”  AND  “Inpatients”[Mesh] OR Inpatient[tiab] OR inpatients[tiab] OR “Hospitalization”[Mesh] OR “Hospital*”[tiab] OR Patient*[tiab] OR "electronic health Record*" OR "Computerised Medical Record*" OR "electronic health record*" OR "electronic medical record*" OR EMR[tiab] OR EHR[tiab] OR "Electronic Health Records"[Mesh] |
| --- | --- |
| **IEEE** | "All Metadata":"acute kidney injury" OR "All Metadata":"AKI" OR "All Metadata":"dialysis") AND ("All Metadata":"machine learning" OR "All Metadata":"artificial intelligence") AND ("All Metadata":"hospital*" OR "All Metadata":"inpatient*") |

IEEE = Institute of Electrical and Electronic Engineers

**Table S2** List of excluded models

| Linear regression |
| --- |
| Logistic regression (stepwise and/or backward and/or discrete time) |
| Naïve Bayes |
| Fuzzy Logic Systems |

PROBAST Forms

Study 1

**PROBAST**

(Prediction model study Risk Of Bias Assessment Tool)

Published in Annals of Internal Medicine (freely available):

1. PROBAST: A Tool to Assess the Risk of Bias and Applicability of Prediction Model Studies

2. PROBAST: A Tool to Assess Risk of Bias and Applicability of Prediction Model Studies: Explanation and Elaboration

**What does PROBAST assess?**

PROBAST assesses both the *risk of bias* and *concerns regarding applicability* of a study that evaluates (develops, validates or updates) a multivariable diagnostic or prognostic prediction model. It is designed to assess primary studies included in a systematic review.

*Bias* occurs if systematic flaws or limitations in the design, conduct or analysis of a primary study distort the results. For the purpose of prediction modelling studies, we have defined *risk of bias* to occur when shortcomings in the study design, conduct or analysis lead to systematically distorted estimates of a model’s predictive performance or to an inadequate model to address the research question. Model predictive performance is typically evaluated using calibration, discrimination and sometimes classification measures, and these are likely inaccurately estimated in studies with high risk of bias. *Applicability* refers to the extent to which the prediction model from the primary study matches your systematic review question, for example in terms of the participants, predictors or outcome of interest.

A primary study may include the development and/or validation or update of more than one prediction model. A PROBAST assessment should be completed for each distinct model that is developed, validated or updated (extended) for making individualised predictions. Where a publication assesses multiple prediction models, only complete a PROBAST assessment for those models that meet the inclusion criteria for your systematic review. Please note that subsequent use of the term “model” includes derivatives of models, such as simplified risk scores, nomograms, or recalibrations of models.

PROBAST is not designed for all multivariable diagnostic or prognostic studies. For example, studies using multivariable models to identify predictors associated with an outcome but not attempting to develop a model for making individualised predictions are not covered by PROBAST.

PROBAST includes four steps.

| **Step** | **Task** | **When to complete** |
| --- | --- | --- |
| **1** | Specify your systematic review question(s) | Once per systematic review |
| **2** | Classify the type of prediction model evaluation | Once for each model of interest in each publication being assessed, for each relevant outcome |
| **3** | Assess risk of bias and applicability | Once for each development and validation of each distinct prediction model in a publication |
| **4** | Overall judgment | Once for each development and validation of each distinct prediction model in a publication |

**Step 1: Specify your systematic review question** State your systematic review question to facilitate the assessment of the applicability of the evaluated models to your question. *The following table should be completed once per systematic review.*

| Criteria | Specify your Systematic Review Question |
| --- | --- |
| *Intended use of model:* | To evaluate the performance and accuracy of externally validated machine learning models in the prediction of AKI in general hospital patients |
| ***Participants*** *including selection criteria and setting:* | We included studies that developed or validated machine learning models for the prediction of AKI (stage 2, 3 or dialysis) in a general hospital population. We excluded studies: (i) in patients under 18 years of age, (ii) which used an outcome definition of AKI other than KDIGO, (iii) presenting models with no external validation and (iv) any comments or editorials or meta-analysis or systematic reviews. |
| ***Predictors*** *(used in prediction modelling), including types of predictors (e.g. history, clinical examination, biochemical markers, imaging tests), time of measurement, specific measurement issues (e.g., any requirements/ prohibitions for specialized equipment):* | All available |
| *Outcome to be predicted:* | Acute Kidney Injury – stage 1, 2,3 or dialysis - defined according to KDIGO criteria. |

**Step 2: Classify the type of prediction model evaluation** Use the following table to classify the evaluation as model development, model validation or model update, or combination. Different signalling questions apply for different types of prediction model evaluation. If the evaluation does not fit one of these classifications, then PROBAST should not be used.

| **Classify the evaluation based on its aim** | | | |
| --- | --- | --- | --- |
| **Type of Prediction Study** | **PROBAST Boxes to Complete** | **Tick as Appropriate** | **Definition for and type of prediction model study** |
| Development only | Development |  | Prediction model development without external validation. These studies may include internal validation methods, such as bootstrapping and cross-validation techniques. |
| Development and Validation | Development and Validation |  | Prediction model development combined with external validation in other participants in the same article. |
| Validation only | Validation |  | External validation of existing (previously developed) model in other participants |

*This table should be completed once for each publication being assessed and for each relevant outcome in your review.*

| Publication Reference | Kim et al. 2021 |
| --- | --- |
| Models of Interest | 2 prediction models created using RNN algorithms and XGBoost |
| Outcome of Interest | AKI - any stage & stage 2 or higher in the next 7 days |

**Step 3: Assess risk of bias and applicability** PROBAST is structured as four key domains. Each domain is judged for risk of bias (low, high or unclear) and includes signalling questions to help make judgements. Signalling questions are rated as yes (Y), probably yes (PY), probably no (PN), no (N) or no information (NI). All signalling questions are phrased so that “yes” indicates absence of bias. Any signalling question rated as “no” or “probably no” flags the potential for bias; you will need to use your judgement to determine whether the domain should be rated as “high”, “low” or “unclear” risk of bias. The guidance document contains further instructions and examples on rating signalling questions and risk of bias for each domain.

The first three domains are also rated for concerns regarding applicability (low/ high/ unclear) to your review question defined above.

*Complete all domains separately for each evaluation of a distinct model. Shaded boxes indicate where signalling questions do not apply and should not be answered.*

| **Domain 1: Participants** | | | |
| --- | --- | --- | --- |
| 1. **Risk of Bias** | | | |
| *Describe the sources of data and criteria for participant selection:*  The study population was sourced from Seoul National University Bundang Hospital and Seoul National University Hospital and included all patients aged 18 and over that were hospitalised for more than 48 hours between 2013 and 2017 (Data obtained from EHRs). The exclusion criteria were (1) no baseline or follow-up creatinine (Cr) measurements, (2) baseline estimated glomerular filtration rate (eGFR) less than 15 mL/min/1.73 m 2 or Cr greater than 4.0 mg/dL or end-stage kidney disease at admission, (3) no other laboratory test results used in the model, (4) no BMI or vital sign measurements, and (5) an AKI diagnosis at admission (day 1) | | | |
|  | | Dev | Val |
| 1.1 Were appropriate data sources used, e.g. cohort, RCT or nested case-control study data? | | PN | PN |
| 1.2 Were all inclusions and exclusions of participants appropriate? | | PN | PN |
| **Risk of Bias introduced by selection of participants** | **Risk:** (high/low/unclear) | High | High |
| *Rationale of bias rating:*  Rationale for 1.1: Data was sourced from EHRs. Routine care registries have higher ROB because data are often collected for a purpose other than development or validation of a prognostic model. Data in these registries is also often inconsistently measured and recorded.  Rationale 1.2 Patients were excluded if they had no BMI, vital signs or “no other lab test result which was included in the model | | | |
| 1. Applicability | | | |
| *Describe included participants, setting and dates:*  The settings were all hospital wards of Seoul National University Bundang Hospital and Seoul National University Hospital (two affiliated but geographically separate hospitals) between 2013 and 2017 | | | |
| **Concern that the included participants and setting do not match the review question** | **Concern:** (high/low/unclear) | Low | Low |
| *Rationale of applicability rating:*  All participants were adult and all hospital wards were considered so the participants and setting match the review question. | | | |

| **Domain 2: Predictors** | | | |
| --- | --- | --- | --- |
| 1. **Risk of Bias** | | | |
| *List and describe predictors included in the final model, e.g. definition and timing of assessment:*  There were 107 variables included in the model (each variable fell under one of the broad categories of demographics, vitals, comorbidities, medications, and clinical conditions). | | | |
|  | | Dev | Val |
| 2.1 Were predictors defined and assessed in a similar way for all participants? | | PY | PY |
| 2.2 Were predictor assessments made without knowledge of outcome data? | | NI | NI |
| 2.3 Are all predictors available at the time the model is intended to be used? | | PY | PY |
| **Risk of Bias introduced by predictors or their assessment** | **Risk:** (high/low/unclear) | Low | Low |
| *Rationale of bias rating:*  Rationale for 2.1: As this includes 2 connected hospitals, one would assume that variables are treated and measured similarly for all patients  Rationale 2.3 The chosen variables predate the outcome chronologically, and therefore should be present. | | | |
| 1. Applicability | | | |
| **Concern that the definition, assessment, or timing of predictors in the model do not match the review question** | **Concern:** (high/low/unclear) | High | High |
| *Rationale of applicability rating:*  An average general hospital is unlikely to produce any degree of patient data completeness for 97 clinical and demographic variables. | | | |

| **Domain 3: Outcomes** | | | |
| --- | --- | --- | --- |
| 1. **Risk of Bias** | | | |
| *Describe the outcome, how it was defined and determined, and the time interval between predictor assessment and outcome determination:*  Any AKI – KDIGO sCr defined – within 7 days of the present | | | |
|  | | Dev | Val |
| 3.1 Was the outcome determined appropriately? | | Y | Y |
| 3.2 Was a pre-specified and standard outcome definition used? | | Y | Y |
| 3.3 Were predictors excluded from the outcome definition? | | N | N |
| 3.4 Was the outcome defined and determined in a similar way for all participants? | | Y | Y |
| 3.5 Was the outcome determined without knowledge of predictor information? | | U | U |
| 3.6 Was the time interval between predictor assessment and outcome determination appropriate? | | PY | PY |
| **Risk of Bias introduced by the outcome or its determination** | **Risk:** (high/low/unclear) | high | high |
| *Rationale of bias rating:*  Rationale for 3.3 and 3.5: SCr was included as a variable and in the outcome definition (incorporation bias) | | | |
| 1. Applicability | | | |
| *At what time point was the outcome determined:*  Within 7 days from the present  *If a composite outcome was used, describe the relative frequency/distribution of each contributing outcome:* | | | |
| **Concern that the outcome, its definition, timing or determination do not match the review question** | **Concern:** (high/low/unclear) | low | low |
| *Rationale of applicability rating:*  KDIGO definition was used and the time period at which the outcome was predicted was appropriate considering our criteria. | | | |

| **Domain 4: Analysis** | | | |
| --- | --- | --- | --- |
| **Risk of Bias** | | | |
| *Describe numbers of participants, number of candidate predictors, outcome events and events per candidate predictor:*  Number of participants: From SNUBH training = 69081 (90%), internal val = 7675 (10%) and for external val from SNUH = 72352.  Number of candidate predictors = 107.  Outcome events = 5.91% in SNUBH = 4536  and 3.63% in SNUH = 2626  EPV SNUBH = 42  SNUH = 24 | | | |
| *Describe how the model was developed (for example in regards to modelling technique (e.g. survival or logistic modelling), predictor selection, and risk group definition):*  Model 1 was a recurrent neural network which had a many-to-one architecture with 7 sequential inputs and 1 prediction output. The outcome variable for model 1 was the occurrence of AKI in the next 7 days. Padding and masking techniques were used for instances with sequences shorter than the length of the sliding window. The padded sequences were not used in training and inference processes. To compare the performance with model 1, we developed an additional gradient boosting model based on the same training data set. The gradient boosting model was trained using the XGBoost algorithm.  For continuous prediction of AKI, the training and validation data sets were organized as multiple sliding windows of features and target labels fed to the input layer of the RNN model (Figure 1A). The  length of the sliding window was selected as 7 days and features up to 2 weeks after admission were utilized. Therefore, all time points were considered for both patients with AKI and non-AKI during the length of stay before AKI occurrence or discharge*.* | | | |
| *Describe whether and how the model was validated, either internally (e.g. bootstrapping, cross validation, random split sample) or externally (e.g. temporal validation, geographical validation, different setting, different type of participants):*  Model was internally validated on a portion of the SNUBH population and then externally validated on the SNUH population | | | |
| *Describe the performance measures of the model, e.g. (re)calibration, discrimination, (re)classification, net benefit, and whether they were adjusted for optimism:*  AUROC  Accuracy, sensitivity, specificity, PPV, NPV  F1 score | | | |
| *Describe any participants who were excluded from the analysis:*  The exclusion criteria were (1) no baseline or follow-up creatinine (Cr) measurements, (2) baseline estimated glomerular filtration rate (eGFR) less than 15 mL/min/1.73 m 2 or Cr greater than 4.0 mg/dL or end-stage kidney disease at admission, (3) no other laboratory test results used in the model, (4) no BMI or vital sign measurements, and (5) an AKI diagnosis at admission (day 1). | | | |
| *Describe missing data on predictors and outcomes as well as methods used for missing data:*  The last observation carried forward method was used for missing values after baseline. | | | |
|  | | Dev | Val |
| 4.1 Were there a reasonable number of participants with the outcome? | | PY | Y |
| 4.2 Were continuous and categorical predictors handled appropriately? | | Y | Y |
| 4.3 Were all enrolled participants included in the analysis? | | NI | NI |
| 4.4 Were participants with missing data handled appropriately? | | PY | PY |
| 4.5 Was selection of predictors based on univariable analysis avoided? | | Y |  |
| 4.6 Were complexities in the data (e.g. censoring, competing risks, sampling of controls) accounted for appropriately?  For continuous prediction of AKI, the training and validation data sets were organized as multiple sliding windows of features and target labels fed to the input layer of the RNN model (Figure 1A). The length of the sliding window was selected as 7 days and features up to 2 weeks after admission were utilized. Therefore, all time points were considered for both patients with AKI and non-AKI during the length of stay before AKI occurrence or discharge | | PY | PY |
| 4.7 Were relevant model performance measures evaluated appropriately? | | PY | PY |
| 4.8 Were model overfitting and optimism in model performance accounted for?  Alpha dropout, L2 regularization, and early stopping approaches were implemented to prevent overfitting*.* | | Y |  |
| 4.9 Do predictors and their assigned weights in the final model correspond to the results from multivariable analysis? | | NI |  |
| **Risk of Bias introduced by the analysis** | **Risk:** (high/low/unclear) | Low | Low |
| *Rationale of bias rating:*  4.1 The entire data set was imbalanced due to the relatively low incidence of in-hospital AKI. Therefore, we  applied a class weight parameter to the loss function to handle class imbalances. Alpha dropout, L2 regularization, and early stopping approaches were implemented to prevent overfitting. | | | |

**Step 4: Overall assessment**

Use the following tables to reach overall judgements about risk of bias and concerns regarding applicability of the prediction model evaluation (development and/or validation) across all assessed domains.

*Complete for each evaluation of a distinct model.*

| **Reaching an overall judgement about risk of bias of the prediction model evaluations** | |
| --- | --- |
| **Low Risk of Bias** | If all domains were rated low risk of bias. If a prediction model was developed without any external validation, and it was rated as low risk of bias for all domains, consider downgrading to high risk of bias. Such a model can only be considered as low risk of bias, if the development was based on a very large dataset and included some form of internal validation. |
| **High Risk of Bias** | If at least one domain is judged to be at high risk of bias. |
| **Unclear Risk of Bias** | If an unclear risk of bias was noted in at least one domain and it was low risk for all other domains |

| **Reaching an overall judgement about applicability of the prediction model evaluation** | |
| --- | --- |
| **Low concerns regarding applicability** | If low concerns regarding applicability for all domains, the prediction model evaluation is judged to have **low concerns regarding applicability**. |
| **High concerns regarding applicability** | If high concerns regarding applicability for at least one domain, the prediction model evaluation is judged to have **high concerns regarding applicability**. |
| **Unclear concerns regarding applicability** | If unclear concerns (but no “high concern”) regarding applicability for at least one domain, the prediction model evaluation is judged to have **unclear concerns regarding applicability** overall. |

| **Overall judgement about risk of bias and applicability of the prediction model evaluation** | | |
| --- | --- | --- |
| **Overall judgement of risk of bias** | **RISK:**  *(low/ high/ unclear)* | **High** |
| *Summary of sources of potential bias:*  High ROB for participants and outcomes | | |
| **Overall judgement of applicability** | **CONCERN:**  *(low/ high/ unclear)* | **High** |
| *Summary of applicability concerns:*  Concerns regarding predictor variables | | |

Study 2

**PROBAST**

(Prediction model study Risk Of Bias Assessment Tool)

Published in Annals of Internal Medicine (freely available):

1. PROBAST: A Tool to Assess the Risk of Bias and Applicability of Prediction Model Studies

2. PROBAST: A Tool to Assess Risk of Bias and Applicability of Prediction Model Studies: Explanation and Elaboration

**What does PROBAST assess?**

PROBAST assesses both the *risk of bias* and *concerns regarding applicability* of a study that evaluates (develops, validates or updates) a multivariable diagnostic or prognostic prediction model. It is designed to assess primary studies included in a systematic review.

*Bias* occurs if systematic flaws or limitations in the design, conduct or analysis of a primary study distort the results. For the purpose of prediction modelling studies, we have defined *risk of bias* to occur when shortcomings in the study design, conduct or analysis lead to systematically distorted estimates of a model’s predictive performance or to an inadequate model to address the research question. Model predictive performance is typically evaluated using calibration, discrimination and sometimes classification measures, and these are likely inaccurately estimated in studies with high risk of bias. *Applicability* refers to the extent to which the prediction model from the primary study matches your systematic review question, for example in terms of the participants, predictors or outcome of interest.

A primary study may include the development and/or validation or update of more than one prediction model. A PROBAST assessment should be completed for each distinct model that is developed, validated or updated (extended) for making individualised predictions. Where a publication assesses multiple prediction models, only complete a PROBAST assessment for those models that meet the inclusion criteria for your systematic review. Please note that subsequent use of the term “model” includes derivatives of models, such as simplified risk scores, nomograms, or recalibrations of models.

PROBAST is not designed for all multivariable diagnostic or prognostic studies. For example, studies using multivariable models to identify predictors associated with an outcome but not attempting to develop a model for making individualised predictions are not covered by PROBAST.

PROBAST includes four steps.

| **Step** | **Task** | **When to complete** |
| --- | --- | --- |
| **1** | Specify your systematic review question(s) | Once per systematic review |
| **2** | Classify the type of prediction model evaluation | Once for each model of interest in each publication being assessed, for each relevant outcome |
| **3** | Assess risk of bias and applicability | Once for each development and validation of each distinct prediction model in a publication |
| **4** | Overall judgment | Once for each development and validation of each distinct prediction model in a publication |

**Step 1: Specify your systematic review question** State your systematic review question to facilitate the assessment of the applicability of the evaluated models to your question. *The following table should be completed once per systematic review.*

| Criteria | Specify your Systematic Review Question |
| --- | --- |
| *Intended use of model:* | To evaluate the performance and accuracy of externally validated machine learning models in the prediction of AKI in general hospital patients |
| ***Participants*** *including selection criteria and setting:* | We included studies that developed or validated machine learning models for the prediction of AKI (stage 2, 3 or dialysis) in a general hospital population. We excluded studies: (i) in patients under 18 years of age, (ii) which used an outcome definition of AKI other than KDIGO, (iii) presenting models with no external validation and (iv) any comments or editorials or meta-analysis or systematic reviews. |
| ***Predictors*** *(used in prediction modelling), including types of predictors (e.g. history, clinical examination, biochemical markers, imaging tests), time of measurement, specific measurement issues (e.g., any requirements/ prohibitions for specialized equipment):* | All available |
| *Outcome to be predicted:* | Acute Kidney Injury – stage 1, 2,3 or dialysis - defined according to KDIGO criteria. |

**Step 2: Classify the type of prediction model evaluation** Use the following table to classify the evaluation as model development, model validation or model update, or combination. Different signalling questions apply for different types of prediction model evaluation. If the evaluation does not fit one of these classifications, then PROBAST should not be used.

| **Classify the evaluation based on its aim** | | | |
| --- | --- | --- | --- |
| **Type of Prediction Study** | **PROBAST Boxes to Complete** | **Tick as Appropriate** | **Definition for and type of prediction model study** |
| Development only | Development |  | Prediction model development without external validation. These studies may include internal validation methods, such as bootstrapping and cross-validation techniques. |
| Development and Validation | Development and Validation |  | Prediction model development combined with external validation in other participants in the same article. |
| Validation only | Validation |  | External validation of existing (previously developed) model in other participants |

*This table should be completed once for each publication being assessed and for each relevant outcome in your review.*

| Publication Reference | Churpek et al. 2020 |
| --- | --- |
| Models of Interest | Machine Learning Risk Score (int and ext validation) |
| Outcome of Interest | Early prediction of KDIGO defined AKI |

**Step 3: Assess risk of bias and applicability** PROBAST is structured as four key domains. Each domain is judged for risk of bias (low, high or unclear) and includes signalling questions to help make judgements. Signalling questions are rated as yes (Y), probably yes (PY), probably no (PN), no (N) or no information (NI). All signalling questions are phrased so that “yes” indicates absence of bias. Any signalling question rated as “no” or “probably no” flags the potential for bias; you will need to use your judgement to determine whether the domain should be rated as “high”, “low” or “unclear” risk of bias. The guidance document contains further instructions and examples on rating signalling questions and risk of bias for each domain.

The first three domains are also rated for concerns regarding applicability (low/ high/ unclear) to your review question defined above.

*Complete all domains separately for each evaluation of a distinct model. Shaded boxes indicate where signalling questions do not apply and should not be answered.*

| **Domain 1: Participants** | | | |
| --- | --- | --- | --- |
| 1. **Risk of Bias** | | | |
| *Describe the sources of data and criteria for participant selection:*  This study included 3 distinct adult (18 years) patient cohorts (retrospective cohort study of prospectively collected data). Participants were sourced from the University of Chicago hospital admissions from 2008-2016 for the internal validation (n = 48 463), and from Loyola University Medical Center (LUMC) from 2007 to 2017 (n = 200 613), and NorthShore University Health System (NUS) from 2006 to 2016 (n = 246 895) for external validation. Participants were excluded if they had nor SCr values recorded during their hospital stay, their first creatinine value was recorded after last vital signs (n = 4,673), if they had no ward/ICU/ED observations between first creatinine and last vital signs (n = 4,798), or if stage 2 AKI occured before first ward/ICU/ED observation (n = 1,269). | | | |
|  | | Int Val | Ext Val |
| 1.1 Were appropriate data sources used, e.g. cohort, RCT or nested case-control study data? | | PN | PN |
| 1.2 Were all inclusions and exclusions of participants appropriate? | | Y | Y |
| **Risk of Bias introduced by selection of participants** | **Risk:** (high/low/unclear) | High | High |
| *Rationale of bias rating:*  Rationale for 1.1: Data was sourced from EHRs. Routine care registries have higher ROB because data are often collected for a purpose other than development or validation of a prognostic model. Data in these registries is also often inconsistently measured and recorded. | | | |
| 1. Applicability | | | |
| *Describe included participants, setting and dates:*  See above. | | | |
| **Concern that the included participants and setting do not match the review question** | **Concern:** (high/low/unclear) | low | low |
| *Rationale of applicability rating:*  Adult patients from general hospital wards were included. | | | |

| **Domain 2: Predictors** | | | |
| --- | --- | --- | --- |
| 1. **Risk of Bias** | | | |
| *List and describe predictors included in the final model, e.g. definition and timing of assessment:*  97 Variables were considered originally and this was narrowed down to 57 for the final model. These predictors included demographic characteristics, vital signs, routine chemistry and hematology laboratory values, trends of vital sign and laboratory values (eg, highest heart rate in previous 24 hours), and nursing documentation (eg, Braden score). The median (for continuous data) or mode (for categorical data) by location was imputed for missing predictor values that remained after carry-forward imputation. | | | |
|  | | Int Val | Ext Val |
| 2.1 Were predictors defined and assessed in a similar way for all participants? | | PY | PY |
| 2.2 Were predictor assessments made without knowledge of outcome data? | | NI | NI |
| 2.3 Are all predictors available at the time the model is intended to be used? | | PY | PY |
| **Risk of Bias introduced by predictors or their assessment** | **Risk:** (high/low/unclear) | Low | Low |
| *Rationale of bias rating:*  Rationale for 2.1: The variables were not explicitly mentioned and defined since there were so many.  Rationale for 2.3: There are a lot of variables included in the model which may make it more likely that not all of them will be available, however, they do mention that they selected predictors specifically for their availability. | | | |
| 1. Applicability | | | |
| **Concern that the definition, assessment, or timing of predictors in the model do not match the review question** | **Concern:** (high/low/unclear) | Low | low |
| *Rationale of applicability rating:*  Overall this is the model with smallest number of predictor variables (59) | | | |

| **Domain 3: Outcomes** | | | |
| --- | --- | --- | --- |
| 1. **Risk of Bias** | | | |
| *Describe the outcome, how it was defined and determined, and the time interval between predictor assessment and outcome determination:*  The outcome predicted was KDIGO SCr-defined stage 2 AKI. Predictors were assessed during hospital admission to predict the probability of the outcome occurring within a 48-hour interval. | | | |
|  | | Int Val | Ext Val |
| 3.1 Was the outcome determined appropriately? | | Y | Y |
| 3.2 Was a pre-specified and standard outcome definition used? | | Y | Y |
| 3.3 Were predictors excluded from the outcome definition? | | N | N |
| 3.4 Was the outcome defined and determined in a similar way for all participants? | | Y | Y |
| 3.5 Was the outcome determined without knowledge of predictor information? | | PN | PN |
| 3.6 Was the time interval between predictor assessment and outcome determination appropriate? | | Y | Y |
| **Risk of Bias introduced by the outcome or its determination** | **Risk:** (high/low/unclear) | high | high |
| *Rationale of bias rating:*  Rationale for 3.3: Change in SCr was used in both the outcome definition and as a variable. Incorporation bias.  Rationale for 3.5: No, since this is a retrospective study cohort. | | | |
| 1. Applicability | | | |
| *At what time point was the outcome determined:*  At the end of the 48 hour interval.  *If a composite outcome was used, describe the relative frequency/distribution of each contributing outcome:*  NA | | | |
| **Concern that the outcome, its definition, timing or determination do not match the review question** | **Concern:** (high/low/unclear) | low | low |
| *Rationale of applicability rating:*  AKI was defined according to KDIGO, which follows our review criteria. | | | |

| **Domain 4: Analysis** | | | |
| --- | --- | --- | --- |
| **Risk of Bias** | | | |
| *Describe numbers of participants, number of candidate predictors, outcome events and events per candidate predictor:*  The number of participants n = 48 463 from UC, n = 200 613 from LUMC, and n = 246 895 from NUS. There were 97 candidate predictors, too many to list individually here or recount the events per predictor. | | | |
| *Describe how the model was developed (for example in regards to modelling technique (e.g. survival or logistic modelling), predictor selection, and risk group definition):*  (model only validated in this paper) | | | |
| *Describe whether and how the model was validated, either internally (e.g. bootstrapping, cross validation, random split sample) or externally (e.g. temporal validation, geographical validation, different setting, different type of participants):*  The model was developed in another study and validated in this paper on the original development set (UC) and external validated on datasets from two different hospitals. | | | |
| *Describe the performance measures of the model, e.g. (re)calibration, discrimination, (re)classification, net benefit, and whether they were adjusted for optimism:*  Discrimination was measured by AUROC, was not adjusted for optimism. Calibration and calibration plots were calculated and displayed (revealing that the models overpredicted risk for the highest decile of patients). | | | |
| *Describe any participants who were excluded from the analysis:*  Participants with no SCr values recorded during their admission. | | | |
| *Describe missing data on predictors and outcomes as well as methods used for missing data:*  Missing data that could impact the outcome (such as lack of SCr values for a participant) led to the exclusion of that participant from the study. Missing data in predictors was imputed in a carry-forward method by using the median (for continuous data) or mode (for categorical data) by location. | | | |
|  | | Dev | Val |
| 4.1 Were there a reasonable number of participants with the outcome? | |  | PY |
| 4.2 Were continuous and categorical predictors handled appropriately? | |  | Y |
| 4.3 Were all enrolled participants included in the analysis? | |  | Y |
| 4.4 Were participants with missing data handled appropriately? | |  | PY |
| 4.5 Was selection of predictors based on univariable analysis avoided? | |  |  |
| 4.6 Were complexities in the data (e.g. censoring, competing risks, sampling of controls) accounted for appropriately? | |  | NI |
| 4.7 Were relevant model performance measures evaluated appropriately? | |  | Y |
| 4.8 Were model overfitting and optimism in model performance accounted for? | |  |  |
| 4.9 Do predictors and their assigned weights in the final model correspond to the results from multivariable analysis? | |  |  |
| **Risk of Bias introduced by the analysis** | **Risk:** (high/low/unclear) |  | Low |
| *Rationale of bias rating:* | | | |

**Step 4: Overall assessment**

Use the following tables to reach overall judgements about risk of bias and concerns regarding applicability of the prediction model evaluation (development and/or validation) across all assessed domains.

*Complete for each evaluation of a distinct model.*

| **Reaching an overall judgement about risk of bias of the prediction model evaluations** | |
| --- | --- |
| **Low Risk of Bias** | If all domains were rated low risk of bias. If a prediction model was developed without any external validation, and it was rated as low risk of bias for all domains, consider downgrading to high risk of bias. Such a model can only be considered as low risk of bias, if the development was based on a very large dataset and included some form of internal validation. |
| **High Risk of Bias** | If at least one domain is judged to be at high risk of bias. |
| **Unclear Risk of Bias** | If an unclear risk of bias was noted in at least one domain and it was low risk for all other domains |

| **Reaching an overall judgement about applicability of the prediction model evaluation** | |
| --- | --- |
| **Low concerns regarding applicability** | If low concerns regarding applicability for all domains, the prediction model evaluation is judged to have **low concerns regarding applicability**. |
| **High concerns regarding applicability** | If high concerns regarding applicability for at least one domain, the prediction model evaluation is judged to have **high concerns regarding applicability**. |
| **Unclear concerns regarding applicability** | If unclear concerns (but no “high concern”) regarding applicability for at least one domain, the prediction model evaluation is judged to have **unclear concerns regarding applicability** overall. |

| **Overall judgement about risk of bias and applicability of the prediction model evaluation** | | |
| --- | --- | --- |
| **Overall judgement of risk of bias** | **RISK:**  *(low/ high/ unclear)* | **High** |
| *Summary of sources of potential bias:* | | |
| **Overall judgement of applicability** | **CONCERN:**  *(low/ high/ unclear)* | **Low** |
| *Summary of applicability concerns:*  Low in all categories | | |

Study 3

**PROBAST**

(Prediction model study Risk Of Bias Assessment Tool)

Published in Annals of Internal Medicine (freely available):

1. PROBAST: A Tool to Assess the Risk of Bias and Applicability of Prediction Model Studies

2. PROBAST: A Tool to Assess Risk of Bias and Applicability of Prediction Model Studies: Explanation and Elaboration

**What does PROBAST assess?**

PROBAST assesses both the *risk of bias* and *concerns regarding applicability* of a study that evaluates (develops, validates or updates) a multivariable diagnostic or prognostic prediction model. It is designed to assess primary studies included in a systematic review.

*Bias* occurs if systematic flaws or limitations in the design, conduct or analysis of a primary study distort the results. For the purpose of prediction modelling studies, we have defined *risk of bias* to occur when shortcomings in the study design, conduct or analysis lead to systematically distorted estimates of a model’s predictive performance or to an inadequate model to address the research question. Model predictive performance is typically evaluated using calibration, discrimination and sometimes classification measures, and these are likely inaccurately estimated in studies with high risk of bias. *Applicability* refers to the extent to which the prediction model from the primary study matches your systematic review question, for example in terms of the participants, predictors or outcome of interest.

A primary study may include the development and/or validation or update of more than one prediction model. A PROBAST assessment should be completed for each distinct model that is developed, validated or updated (extended) for making individualised predictions. Where a publication assesses multiple prediction models, only complete a PROBAST assessment for those models that meet the inclusion criteria for your systematic review. Please note that subsequent use of the term “model” includes derivatives of models, such as simplified risk scores, nomograms, or recalibrations of models.

PROBAST is not designed for all multivariable diagnostic or prognostic studies. For example, studies using multivariable models to identify predictors associated with an outcome but not attempting to develop a model for making individualised predictions are not covered by PROBAST.

PROBAST includes four steps.

| **Step** | **Task** | **When to complete** |
| --- | --- | --- |
| **1** | Specify your systematic review question(s) | Once per systematic review |
| **2** | Classify the type of prediction model evaluation | Once for each model of interest in each publication being assessed, for each relevant outcome |
| **3** | Assess risk of bias and applicability | Once for each development and validation of each distinct prediction model in a publication |
| **4** | Overall judgment | Once for each development and validation of each distinct prediction model in a publication |

**Step 1: Specify your systematic review question** State your systematic review question to facilitate the assessment of the applicability of the evaluated models to your question. *The following table should be completed once per systematic review.*

| Criteria | Specify your Systematic Review Question |
| --- | --- |
| *Intended use of model:* | To evaluate the performance and accuracy of machine learning models in the prediction of AKI in general hospital patients |
| ***Participants*** *including selection criteria and setting:* | We included studies that developed or validated machine learning models for the prediction of AKI (stage 2, 3 or dialysis) in a general hospital population. We excluded studies: (i) in patients under 18 years of age, (ii) which used an outcome definition of AKI other than KDIGO, (iii) presenting models with no external validation and (iv) any comments or editorials or meta-analysis or systematic reviews. |
| ***Predictors*** *(used in prediction modelling), including types of predictors (e.g. history, clinical examination, biochemical markers, imaging tests), time of measurement, specific measurement issues (e.g., any requirements/ prohibitions for specialized equipment):* | All available |
| *Outcome to be predicted:* | Acute Kidney Injury – stage 1, 2,3 or dialysis - defined according to KDIGO criteria. |

**Step 2: Classify the type of prediction model evaluation** Use the following table to classify the evaluation as model development, model validation or model update, or combination. Different signalling questions apply for different types of prediction model evaluation. If the evaluation does not fit one of these classifications, then PROBAST should not be used.

| **Classify the evaluation based on its aim** | | | |
| --- | --- | --- | --- |
| **Type of Prediction Study** | **PROBAST Boxes to Complete** | **Tick as Appropriate** | **Definition for and type of prediction model study** |
| Development only | Development |  | Prediction model development without external validation. These studies may include internal validation methods, such as bootstrapping and cross-validation techniques. |
| Development and Validation | Development and Validation | x | Prediction model development combined with external validation in other participants in the same article. |
| Validation only | Validation |  | External validation of existing (previously developed) model in other participants |

*This table should be completed once for each publication being assessed and for each relevant outcome in your review.*

| Publication Reference | Song et al |
| --- | --- |
| Models of Interest | Gradient Boosting with decision trees implemented in a discrete-time survival framework (DS-GBT), LASSO model |
| Outcome of Interest | Any AKI within 48 hrs |

**Step 3: Assess risk of bias and applicability** PROBAST is structured as four key domains. Each domain is judged for risk of bias (low, high or unclear) and includes signalling questions to help make judgements. Signalling questions are rated as yes (Y), probably yes (PY), probably no (PN), no (N) or no information (NI). All signalling questions are phrased so that “yes” indicates absence of bias. Any signalling question rated as “no” or “probably no” flags the potential for bias; you will need to use your judgement to determine whether the domain should be rated as “high”, “low” or “unclear” risk of bias. The guidance document contains further instructions and examples on rating signalling questions and risk of bias for each domain.

The first three domains are also rated for concerns regarding applicability (low/ high/ unclear) to your review question defined above.

*Complete all domains separately for each evaluation of a distinct model. Shaded boxes indicate where signalling questions do not apply and should not be answered.*

| **Domain 1: Participants** | | | |
| --- | --- | --- | --- |
| 1. **Risk of Bias** | | | |
| *Describe the sources of data and criteria for participant selection:*  Six participating GPC (Greater Plain Collaborative) sites in 5 US states from the PCORnet Clinical Data Research Network  Inclusions: Patients 18 - 90 years of age, hospitalized for at least 2 days, with at least 2 sCr from 2010 to the end of 2018. Exclusions: 1) severe CKD as evidence by an eGFR of < 15 ml/min or on RRT prior to the admission, 2) required RRT within 48 hrs of their first documented sCr measurement, 3) Burn patients | | | |
|  | | Dev | Val |
| - 1. Were appropriate data sources used, e.g. cohort, RCT or nested case-control study data?   *PCORnet developed the CDM to support federated research networks by centering its schema on the patient entity, and enforcing data mapping to controlled vocabularies such as Current Procedural Terminology (CPT), SNOMED CT, Healthcare Common Procedure Coding System (HCPCS), the ICD versions 9 and 10, Logical Observation Identifiers Names and Codes (LOINC), and RxNorm15. Using the CDM at all GPC institutions/sites that pass quarterly data quality assessments allows efficient query and analysis execution across different instances of the data model* | | PN | PN |
| 1.2 Were all inclusions and exclusions of participants appropriate? | | PY | PY |
| **Risk of Bias introduced by selection of participants** | **Risk:** (high/low/unclear) | Unclear | unclear |
| *Rationale of bias rating:*  Risk is unclear since Hospital EHR data is not collected with the purpose of being used for prediction models. However, the PCORnet CDM may be the most standardized format and language to harmonize digital health data from different sites. | | | |
| 1. Applicability | | | |
| *Describe included participants, setting and dates:*  Patients 18 - 90 years of age, hospitalized for at least 2 days, with at least 2 sCr from 2010 to the end of 2018 from 6 tertiary hospital in 5 states of US. 153,821 eligible encounters were sourced | | | |
| **Concern that the included participants and setting do not match the review question** | **Concern:** (high/low/unclear) | Low | Low |
| *Rationale of applicability rating:*  Participants are from large tertiary hospital, general ward settings | | | |
|  | | | |

| **Domain 2: Predictors** | | | |
| --- | --- | --- | --- |
| 1. **Risk of Bias** | | | |
| *List and describe predictors included in the final model, e.g. definition and timing of assessment:*  Initial feature set contained > 30,000 distinct features. They then identified 1933 unique variables common to all 6 sites. This included: General demographic details (i.e., age, gender, and race), all  structured clinical variables that are currently supported by PCORnet CDM Version 4, including diagnoses (ICD-9 and ICD-10 codes), procedures (ICD and CPT codes), lab tests (LOINC codes), medications (RXNORM and NDC codes), as well as selected vital signs (e.g., blood pressure, height, weight, BMI)29. All variables are time-stamped and every patient in the dataset was represented by a sequence of clinical events construed by clinical observation vectors aggregated on daily basis (Extended Fig. 1), so that the feature set formed by data prior to or on day t could be used to predict AKI within [t+ 1, t + 2] days for 48-h prediction (or within the next day for 24-h prediction). | | | |
|  | | Dev | Val |
| 2.1 Were predictors defined and assessed in a similar way for all participants? | | PY | PY |
| 2.2 Were predictor assessments made without knowledge of outcome data? | | PY | PY |
| 2.3 Are all predictors available at the time the model is intended to be used? | | PY | PY |
| **Risk of Bias introduced by predictors or their assessment** | **Risk:** (high/low/unclear) | Low | Low |
| *Rationale of bias rating:*  All criteria met | | | |
| 1. Applicability | | | |
| **Concern that the definition, assessment, or timing of predictors in the model do not match the review question** | **Concern:** (high/low/unclear) | High | High |
| *Rationale of applicability rating:*  An average general hospital is unlikely to have any degree in data completeness for 1933 clinical variables | | | |

| **Domain 3: Outcomes** | | | |
| --- | --- | --- | --- |
| 1. **Risk of Bias** | | | |
| *Describe the outcome, how it was defined and determined, and the time interval between predictor assessment and outcome determination:*  Any AKI, defined by KDIGO sCr based criteria, within 48 hrs | | | |
|  | | Dev | Val |
| 3.1 Was the outcome determined appropriately? | | Y | Y |
| 3.2 Was a pre-specified and standard outcome definition used? | | Y | Y |
| 3.3 Were predictors excluded from the outcome definition? | | PN | PN |
| 3.4 Was the outcome defined and determined in a similar way for all participants? | | Y | Y |
| 3.5 Was the outcome determined without knowledge of predictor information? | | U | U |
| 3.6 Was the time interval between predictor assessment and outcome determination appropriate? | | Y | Y |
| **Risk of Bias introduced by the outcome or its determination** | **Risk:** (high/low/unclear) | Low | Low |
| *Rationale of bias rating:*  Differently from other studies, in this one, a second model was trained without values of sCr and BUN to predict AKI. | | | |
| 1. Applicability | | | |
| *At what time point was the outcome determined:*  48 hrs  *If a composite outcome was used, describe the relative frequency/distribution of each contributing outcome:* | | | |
| **Concern that the outcome, its definition, timing or determination do not match the review question** | **Concern:** (high/low/unclear) | Low | Low |
| *Rationale of applicability rating:* | | | |

| **Domain 4: Analysis** | | | |
| --- | --- | --- | --- |
| **Risk of Bias** | | | |
| *Describe numbers of participants, number of candidate predictors, outcome events and events per candidate predictor:*  153,821 pt encounters  1933 predictors  Outcome: Any AKI: 23,267 = 15.1% from full cohort  EPV: 12  NO information on AKI incidence in development vs validation cohorts | | | |
| *Describe how the model was developed (for example in regards to modelling technique (e.g. survival or logistic modelling), predictor selection, and risk group definition):*  We chose Gradient Boosting Tree-based Machines (GBT) as the learning model and then combined it with a discrete-time survival framework using independent censoring. | | | |
| *Describe whether and how the model was validated, either internally (e.g. bootstrapping, cross validation, random split sample) or externally (e.g. temporal validation, geographical validation, different setting, different type of participants):*  For each of the six prediction tasks (24 or 48-h prediction of the 3 AKI stages), we repeated the following steps for model training, validating/ transporting, model refitting, and post-analysis: (a) we first split both source and target data into derivation, calibration, internal validation and temporal validation sets, with calibration and internal validation being 15% of randomly held-out inpatient encounters occurred between 1 January 2010 and 31 December 2016 and temporal validation occurred after 1 January 2017; (b) at the training stage, we tuned a set of hyperparameters (learning rate, number of trees, depth of trees,  sample rates, and number of cases in leave notes) with Bayesian optimization approach using fivefold cross validation and retrained a final reference model using all training data (source); (c) at the validation stage, we re-calibrated the model on the calibration source data set and then evaluated the model performance on both the internal and temporal validation sets within source data, as well as the external validation set within target data; (d) at the refitting stage, we iteratively repeated (a) through (c) with models developed on training data from each target site and validated on corresponding validation sets | | | |
| *Describe the performance measures of the model, e.g. (re)calibration, discrimination, (re)classification, net benefit, and whether they were adjusted for optimism:*  AUROC, AUPRC, Calibration  AUROC done in the Int and ext val cohorts w/ all features or w/o sCr and BUN. It was then stratified to different ages, leves of sCr and longer prediction periods | | | |
| *Describe any participants who were excluded from the analysis:*  Exclusions: 1) severe CKD as evidence by an eGFR of < 15 ml/min or on RRT prior to the admission, 2) required RRT within 48 hrs of their first documented sCr measurement, 3) Burn patients | | | |
| *Describe missing data on predictors and outcomes as well as methods used for missing data:*  Sample-and-hold - carried earlier available observation over. | | | |
|  | | Dev | Val |
| 4.1 Were there a reasonable number of participants with the outcome? | | PN | PN |
| 4.2 Were continuous and categorical predictors handled appropriately? | | Y | Y |
| 4.3 Were all enrolled participants included in the analysis? | | Y | Y |
| 4.4 Were participants with missing data handled appropriately? | | Y | Y |
| 4.5 Was selection of predictors based on univariable analysis avoided? | | Y |  |
| 4.6 Were complexities in the data (e.g. censoring, competing risks, sampling of controls) accounted for appropriately? | | Y | Y |
| 4.7 Were relevant model performance measures evaluated appropriately? | | Y | Y |
| 4.8 Were model overfitting and optimism in model performance accounted for?  Model overfitting was accounted for | | Y |  |
| 4.9 Do predictors and their assigned weights in the final model correspond to the results from multivariable analysis? | | U |  |
| **Risk of Bias introduced by the analysis** | **Risk:** (high/low/unclear) | Low | Low |
| *Rationale of bias rating:*  4.1 The EPV is 12 which is low. The number of participants is Iow for the number of predictors used in the model which may lead to overfitting. However, model overfitting is accounted for. | | | |

**Step 4: Overall assessment**

Use the following tables to reach overall judgements about risk of bias and concerns regarding applicability of the prediction model evaluation (development and/or validation) across all assessed domains.

*Complete for each evaluation of a distinct model.*

| **Reaching an overall judgement about risk of bias of the prediction model evaluations** | |
| --- | --- |
| **Low Risk of Bias** | If all domains were rated low risk of bias. If a prediction model was developed without any external validation, and it was rated as low risk of bias for all domains, consider downgrading to high risk of bias. Such a model can only be considered as low risk of bias, if the development was based on a very large dataset and included some form of internal validation. |
| **High Risk of Bias** | If at least one domain is judged to be at high risk of bias. |
| **Unclear Risk of Bias** | If an unclear risk of bias was noted in at least one domain and it was low risk for all other domains |

| **Reaching an overall judgement about applicability of the prediction model evaluation** | |
| --- | --- |
| **Low concerns regarding applicability** | If low concerns regarding applicability for all domains, the prediction model evaluation is judged to have **low concerns regarding applicability**. |
| **High concerns regarding applicability** | If high concerns regarding applicability for at least one domain, the prediction model evaluation is judged to have **high concerns regarding applicability**. |
| **Unclear concerns regarding applicability** | If unclear concerns (but no “high concern”) regarding applicability for at least one domain, the prediction model evaluation is judged to have **unclear concerns regarding applicability** overall. |

| **Overall judgement about risk of bias and applicability of the prediction model evaluation** | | |
| --- | --- | --- |
| **Overall judgement of risk of bias** | **RISK:**  *(low/ high/ unclear)* | **low** |
| *Summary of sources of potential bias:*  Low or unclear in all categories | | |
| **Overall judgement of applicability** | **CONCERN:**  *(low/ high/ unclear)* | **low** |
| *Summary of applicability concerns:*  Low in all categories | | |
